# The HPV16E7 Affibody as a Novel Potential Therapeutic Agent for Treating Cervical Cancer Is Likely Internalized through Dynamin and Caveolin-1 Dependent Endocytosis

**DOI:** 10.3390/biom12081114

**Published:** 2022-08-12

**Authors:** Qingyuan Zhang, Hua Zhu, Zhouying Cui, Yuxiao Li, Jiaying Zhuo, Jingwei Ye, Zhihui Zhang, Zheng Lian, Qianqian Du, Kong-Nan Zhao, Lifang Zhang, Pengfei Jiang

**Affiliations:** 1 Institute of Molecular Virology and Immunology, Department of Microbiology & Immunology, School of Basic Medical Sciences, Wenzhou Medical University, Wenzhou 325035, China; 2 Department of Gynecology, The First Affiliated Hospital of Wenzhou Medical University, Wenzhou 325035, China; 3 Australian Institute for Bioengineering and Nanotechnology, The University of Queensland, St Lucia 4067, Australia

**Keywords:** affibody, intracellular target proteins, endocytosis, cell cycle arrest, inhibition of cell proliferation

## Abstract

Affibodies targeting intracellular proteins have a great potential to function as ideal therapeutic agents. However, little is known about how the affibodies enter target cells to interact with intracellular target proteins. We have previously developed the HPV16E7 affibody (Z_HPV16E7_384) for HPV16 positive cervical cancer treatment. Here, we explored the underlying mechanisms of Z_HPV16E7_384 and found that Z_HPV16E7_384 significantly inhibited the proliferation of target cells and induced a G1/S phase cell cycle arrest. Furthermore, Z_HPV16E7_384 treatment resulted in the upregulation of retinoblastoma protein (Rb) and downregulation of phosphorylated Rb (pRb), E2F1, cyclin D1, and CDK4 in the target cells. Moreover, treatment with dynamin or the caveolin-1 inhibitor not only significantly suppressed the internalization of Z_HPV16E7_384 into target cells but also reversed the regulation of cell cycle factors by Z_HPV16E7_384. Overall, these results indicate that Z_HPV16E7_384 was likely internalized specifically into target cells through dynamin- and caveolin-1 mediated endocytosis. Z_HPV16E7_384 induced the cell cycle arrest in the G1/S phase at least partially by interrupting HPV16E7 binding to and degrading Rb, subsequently leading to the downregulation of E2F1, cyclin D1, CDK4, and pRb, which ultimately inhibited target cell proliferation. These findings provide a rationale of using Z_HPV16E7_384 to conduct a clinical trial for target therapy in cervical cancer.

## 1. Introduction

Affibodies, a class of small affinity proteins developed from staphylococcal protein A (SPA), have been used for imaging and diagnosis for more than two decades [1,2,3]. Compared to antibodies, the most successful targeted anti-cancer drugs in recent years, affibodies have several advantages including a smaller size, more stability, and faster folding structure [4]. In addition, affibodies can be conjugated with toxins or radioactive substances [4]. Therefore, affibodies and their derivatives have a great potential to function as ideal targeted therapeutic agents.

By now, over 400 studies have reported that more than 40 different targets have been used to select specific affibodies for a variety of applications [4]. Several therapeutic affibodies have now entered preclinical and clinical trials for cancer treatment [5]. In more recent studies, several affibodies that target intracellular proteins show great potential in cancer and other disease therapy, which include the affibodies targeting HPV16 E6 or E7 for cervical cancer, those targeting the EBV LMP1 C-terminal domain or LMP2A N-terminal domain for nasopharyngeal carcinoma, and those targeting Chlamydia trachomatis MOMP for Chlamydia trachomatis [6,7,8,9,10,11]. However, a key question that remains to be answered is whether and how the affibodies enter the target cells to interact with intracellular target proteins, leading to the inhibition of the target cell proliferation and finally to destroy the target cells.

Cervical cancer, the fourth most common cause of cancer death for women, is mainly caused by infection with high-risk human papillomavirus (HR-HPV) such as HPV16 and HPV18 [12,13]. In particular, HPV16 positive cervical cancer accounts for about 50% in all cases [14,15]. Even though cervical cancer has been effectively prevented by commercial HPV vaccines in developed countries, women who have already been infected by HR-HPV and developed HPV associated lesions, especially in developing countries, have to receive the common clinical treatments including surgery, radiation therapy, and chemotherapy, which are invasive with side effects [16]. Therefore, a non-invasive therapy for cervical cancer is urgently needed.

HPV16 E7 causes cellular transformation by targeting pRb, thus leading to cervical carcinogenesis. Therefore, HPV16 E7 is one of the most important onco-proteins in cervical cancer [17]. Z_HPV16E7_384 is an affibody that was generated for the imaging and treatment of HPV16 positive cervical cancer by using HPV16E7 as a target protein through a phage-display platform [6]. Z_HPV16E7_384 can be specifically internalized into HPV16 positive target cells and significantly inhibit HPV16 positive tumor growth in nude mice [6,7]. Even though Z_HPV16E7_384 has a great potential in antitumor therapy, the mechanism of Z_HPV16E7_384 internalized by target cells and that of its anti-tumor effect remain unknown. In this study, we aimed to explore these underlying mechanisms of Z_HPV16E7_384 to lay a foundation for further clinical trials.

## 2. Materials and Methods

### 2.1. Cell Culture

SiHa (ATCC: HTB-35, HPV16 positive), CaSki (ATCC: CRL-1550, HPV16 positive), and C666-1 (ATCC: CVCL_7949, used as HPV16 negative control cell line) were obtained from the American Type Culture Collection and cultured as previously described [6,18].

### 2.2. Preparation of Z_HPV16E7_384

Z_HPV16E7_384 was prepared according to our previous study [6]. Briefly, E. coli BL21 (DE3) transformed with recombinant vector pET21a(+)-Z_HPV16E7_384 was induced by IPTG to express Z_HPV16E7_384 with six His-tags. The Z_HPV16E7_384 protein was purified by affinity chromatography using a precharged Ni-NTA Sepharose column. Wild type affibody Z_WT_ was prepared as the control.

### 2.3. Preparation of FITC-Labelled Z_HPV16E7_384

The HOOK (TM) Dye Labelling Kit (FITC) (Sangon Biotech, Shanghai, China) was used to label FITC fluorescein to Z_HPV16E7_384 and Z_WT_ as per the manufacturer’s instructions. The labelled affibodies (FITC-Z_HPV16E7_384 and FITC-Z_WT_) were stored at −80 °C in the dark.

### 2.4. Indirect Immunofluorescence Assay

The affibody binding to target cells was detected by the indirect immunofluorescence assay (IFA) as described in a previous study [7]. Briefly, after treatment with afiibodies for the indicated time periods, the cells were washed with PBS and fixed with 4% paraformaldehyde for 10 min at room temperature. Then, the cells were washed with cold 0.01 M PBST, and placed in blocking buffer (PBS containing 5% FBS) at 4 °C overnight, followed by incubation with indicated primary antibodies for 1 h at room temperature. The cells were washed with cold PBST three times and incubated with the indicated secondary antibodies for 1 h at room temperature. Cell nuclei were stained with 6 μg/mL of Hoechst33342 (Invitrogen, CA, USA) for 10 min. The cells were analyzed by a confocal fluorescence microscope (TC-1, Nikon, Tokyo, Japan).

### 2.5. Free Z_HPV16E7_384 Blocking Assay

The SiHa cells were first incubated with 50 μm of free Z_HPV16E7_384 for 0, 0.5, 1, 2, 4, and 6 h, respectively. After being washed with PBS, the cells were then incubated with 50 μm of FITC-Z_HPV16E7_384 for 6 h. The SiHa cells incubated only with FITC-Z_HPV16E7_384 were used as a positive control while the SiHa cells incubated with the FITC-Z_WT_ and C666-1 cells incubated with FITC-Z_HPV16E7_384 were used as the negative controls. After being washed with PBS and fixed with 4% paraformaldehyde for 15 min, the cells were stained with 6 μg/mL of Hoechst33342 (Invitrogen, CA, USA) for 10 min. The cells were analyzed by a confocal fluorescence microscope (TC-1, Nikon, Tokyo, Japan). The intensity of fluorescence was analyzed by using the method in previous studies [19,20].

### 2.6. Analysis of Z_HPV16E7_384 Amino Acid Sequence

The amino acid sequence of Z_HPV16E7_384 was aligned with that of SPA (Genbank No. P02976.3) by using the Protein BLAST software (https://blast.ncbi.nlm.nih.gov/Blast.cgi) (accessed on 7 May 2018). The same amino acids between Z_HPV16E7_384 and SPA were shown as dots.

### 2.7. Analysis of the Effect of Fetal Bovine Serum on Z_HPV16E7_384 Internalization

The SiHa cells were seeded in 24-well plates at a density of 1 × 10^5^ cells per well. After 24 h, cells were washed with PBS three times and then incubated with 50 μm of Z_HPV16E7_384 diluted in a cell culture medium supplemented with or without 10% fetal bovine serum (FBS) for 6 h. IFA was performed to analyze the entry of Z_HPV16E7_384 into cells. SiHa cells incubated with Z_WT_ and C666-1 cells incubated with Z_HPV16E7_384 were used as the negative controls. The intensity of fluorescence was analyzed by using the method in previous studies [19,20]. 

### 2.8. HPV16E7 Antibody Blocking Assay

The distribution of the HPV16E7 protein in the SiHa cells was analyzed by IFA according to a previous study [21]. The SiHa and CaSki cells were first incubated with rabbit anti-HPV16E7 polyclonal antibody for 1 h, followed by incubation with 50 μm of Z_HPV16E7_384 for 0.5, 1, 2, and 6 h, respectively. Then, IFA was performed to analyze the entry of Z_HPV16E7_384 into the cells. The intensity of the fluorescence was analyzed by using the method in previous studies [19,20].

### 2.9. Western Blot

Western blot was performed according to our previous study [7]. Briefly, whole cell lysates were run on a 12% SDS-polyacrylamide gel and transferred onto polyvinylidene difluoride membranes (Millipore, MA, USA). Membranes were blocked with 10% skim milk in PBST (1 × PBS + 0.1% Tween-20) for 2 h, incubated with the indicated primary antibodies (diluted at 1:1000) for 2 h at room temperature, and then incubated with the HRP-conjugated secondary antibody (diluted at 1:10,000) for 1 h at room temperature. The protein bands were visualized using 0.005% (*w*/*v*) 4-chloro-1-naphthol and a 0.015% (*v*/*v*) hydrogen peroxidase color development substrate.

### 2.10. Endocytosis Inhibition Tests

The SiHa and CaSki cells were first treated with 30 μm of the CDE inhibitor CPZ, or 100 μm of the DDE inhibitor Dynasore, or 30 μm of the micropinocytosis inhibitor Wortmannin, or 2 mm of the CaDE inhibitor methyl-β-cyclodextrin (MBC), followed by incubation with 50 μm of Z_HPV16E7_384 for 6 h. IFA was performed to detect the internalized Z_HPV16E7_384.

### 2.11. Cell Viability Assay

The SiHa, CaSki, and C666-1 cells were plated onto a 96-well plate at a density of 1 × 10^4^ cells per well, followed by treatment with 30 μm of Z_HPV16E7_384 for 48 h. Then, a cell viability assay was performed with the CCK-8 Kit (Dojindo, Kumamoto, Japan) using the method in the previous study [7]. Cells treated with Z_WT_ or PBS were used as the negative controls while cells treated with 10 mm of hydroxyurea were used as the positive control.

### 2.12. Colony Formation Assay

The SiHa, CaSki, and C666-1 cells were respectively seeded in 6-well plates at a density of 2000 cells per well and exposed to 30 μm of Z_HPV16E7_384 in complete media for 14 days. The colonies were fixed by 4% paraformaldehyde for 15 min and stained with crystal violet staining solution (Beyotime Biotechnology, Shanghai, China) for 10 min and washed with ultrapure water three times. Visible colonies were photographed by a Molecular Imager Gel Do XR+ System (Bio-Rad, CA, USA) and counted using ImageJ software (NIH, Bethesda, MD, USA). SiHa, CaSki, and C666-1 cells treated with Z_WT_ or PBS were used as the controls.

### 2.13. EdU Proliferation Assay

The SiHa, CaSki, or C666-1 cells were respectively seeded in 24-well plates at a density of 1 × 10^5^ cells per well. After 24 h, the cells were exposed to 30 μm of Z_HPV16E7_384 in complete media for 48 h. Cell proliferation was detected by the incorporation of 5-ethynyl-2′-deoxyuridine (EdU) with an EDU Cell Proliferation Assay Kit (Beyotime Biotechnology, Shanghai, China) according to the manufacturer’s protocol. Cell nuclei were stained with 6 μg/mL of Hoechst33342 (Invitrogen, CA, USA) for 10 min. The proportion of cells incorporating EDU was determined by confocal fluorescence microscope (TC-1, Nikon, Tokyo, Japan). The SiHa, CaSki, and C666-1 cells treated with Z_WT_ or PBS were used as the negative controls while the SiHa, CaSki, and C666-1 cells treated with 10 mm of hydroxyurea were used as the positive controls.

### 2.14. Flow Cytometry

The SiHa, CaSki, and C666-1 cells were respectively seeded in 6-well plates at a density of 5 × 10^5^ cells per well. After 24 h, the cells were treated with 30 μm of Z_HPV16E7_384 for 48 h. Then, the DNA contents of cells were measured using a Cell Cycle Detection Kit (KeyGen BioTECH, Nanjing, China). Data were acquired with a FACScalibur (Becton Dickinson, NJ, USA) flow cytometry system. Cell cycle distributions were calculated using FlowJo10.6.2 software (Becton Dickinson, NJ, USA). The SiHa, CaSki, and C666-1 cells treated with Z_WT_ or PBS were used as the controls.

### 2.15. Statistical Analysis

All experiments were performed at least three times using different cell preparations. The data from the representative experiments are shown in the figures. Calculation of the means and standard deviations (SD) and statistical analysis were performed with SPSS 17.0. The significance of the differences among treatments was determined using one-way analysis of variance, with a *p*-value below 0.05 considered statistically significant.

## 3. Results

### 3.1. Z_HPV16E7_384 Was Successfully Prepared

Z_HPV16E7_384 and Z_WT_ were expressed and purified according to the previous study [6] and confirmed by SDS-PAGE and Western blot (Figure S1).

### 3.2. Z_HPV16E7_384 Specifically Bound to HPV16 Positive Cervical Cancer Cells

**Figure 1:**
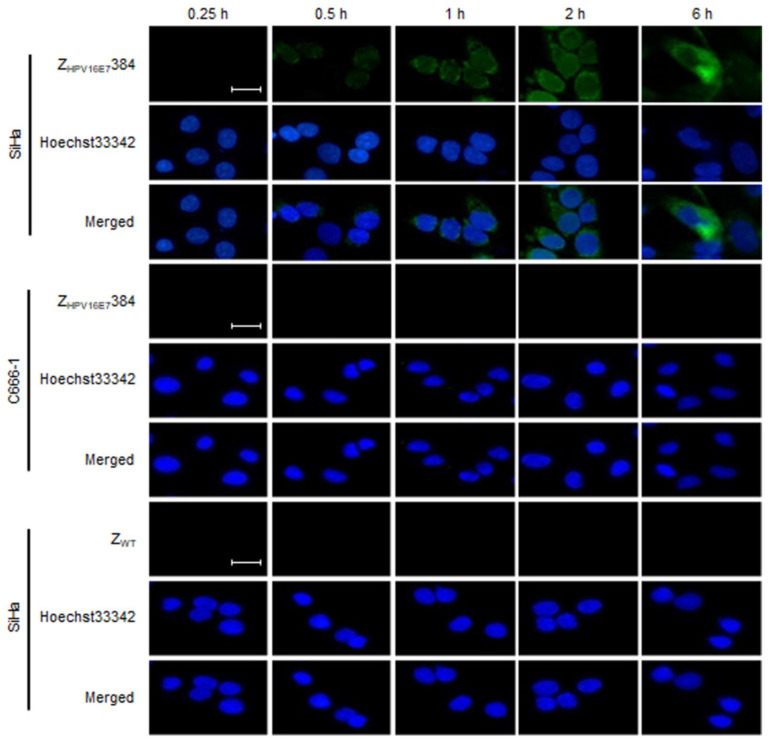
Z_HPV16E7_384 specifically bound to the HPV 16 E7 positive cervical cancer cells. IFA was performed to detect Z_HPV16E7_384 by using mouse anti-His tag monoclonal antibody as the primary antibody and FITC-conjugated goat anti-mouse IgG polyclonal antibody as the secondary antibody (Green). Cell nuclei were counterstained with Hoechst33342 (Blue). Scale bar = 20 μm. The data shown are representative of three independent experiments.

To assess the specificity of Z_HPV16E7_384 binding to the HPV16 positive cervical cancer cells, the SiHa cells pre-treated with 50 μm of Z_HPV16E7_384 for 0.25, 0.5, 1, 2, or 6 h, respectively, were analyzed by IFA. The SiHa cells pre-treated with 50 μm of Z_WT_ and C666-1 cells pre-treated with 50 μm of Z_HPV16E7_384 were used as the negative controls. As shown in Figure 1, specific green fluorescence signals were detected in the SiHa cells after being treated with Z_HPV16E7_384 for 0.5 h, whereas no positive signals were observed in the control cells over the time period.

### 3.3. Z_HPV16E7_384 Significantly Suppressed the Proliferation of HPV16 Positive Cervical Cancer Cells

**Figure 2:**
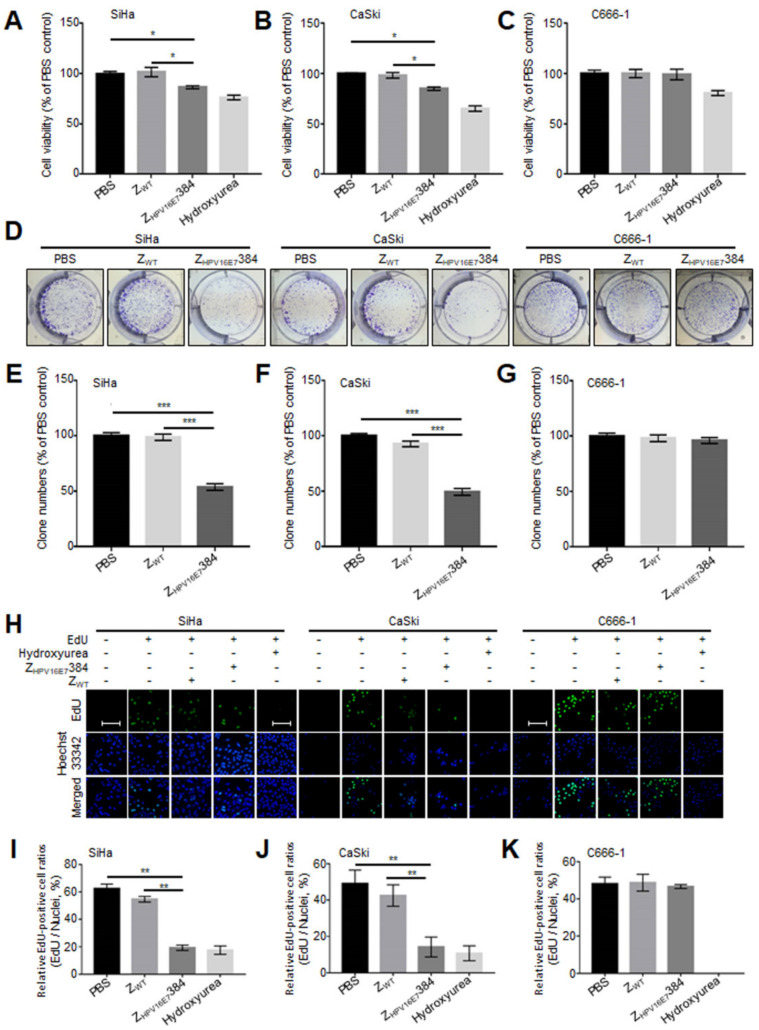
Z_HPV16E7_384 significantly inhibited the proliferation of the target cells (SiHa and Caski) (**A**–**C**) A cell viability assay was performed to evaluate the effect of Z_HPV16E7_384 on the target cells. Cells treated with Z_WT_ or PBS were used as the negative controls while those treated with hydroxyurea were used as the positive controls. (**D**) A colony formation assay was performed to evaluate the suppression of cell proliferation by Z_HPV16E7_384. (**E**–**G**) The clone numbers in (**D**) were counted. (**H**) An EdU proliferation assay was performed to further evaluate the suppression of cell proliferation by Z_HPV16E7_384. Cell proliferation was determined by the incorporation of EdU (green). Cell nuclei were counterstained with Hoechst 33342 (Blue). Scale bar = 100 μm. (**I**–**K**) The ratio of EdU-incorporating live cells was calculated. The data shown are representative of three independent experiments. All experiments were performed in triplicate and data are expressed as the means ± SD (*n* = 3). * *p* < 0.05, ** *p* < 0.01, *** *p* < 0.001.

Z_HPV16E7_384 showed a great anti-cervical cancer efficacy in tumor-bearing nude mice [7]. To confirm the anti-cervical cancer efficacy of Z_HPV16E7_384 in vitro, SiHa, CaSki, and C666-1 cells were treated with Z_HPV16E7_384, followed by the cell viability assay. As shown in Figure 2A–C, the cell viability of the SiHa and CaSki cells treated with Z_HPV16E7_384 significantly decreased compared to those treated with Z_WT_ or PBS. In contrast, the C666-1 cells treated with Z_HPV16E7_384, Z_WT_, or PBS remained fully viable. The data suggest that Z_HPV16E7_384 specifically inhibited the viability of HPV16 positive cervical cancer cells.

Colony formation and EdU assays were then performed to investigate whether Z_HPV16E7_384 suppressed the proliferation of the target cells. As shown in Figure 2D–G, Z_HPV16E7_384 treatment significantly reduced colony formation in the SiHa and CaSki cells whereas the same results were not observed in the SiHa and CaSki cells treated with Z_WT__,_ nor in the C666-1 cells treated with Z_HPV16E7_384. Furthermore, the EdU assay showed that the percentage of EdU-incorporating live cells was significantly decreased in the SiHa and CaSki cells treated with Z_HPV16E7_384 compared to the controls (Figure 2H–K). The results further indicated that Z_HPV16E7_384 profoundly inhibited the proliferation of the HPV16 positive cervical cancer cells.

### 3.4. Z_HPV16E7_384 Induced G1/S Cell Cycle Arrest through Inhibition of the E7/Rb/E2F1/Cyclin D1/CDK4 Pathway

**Figure 3:**
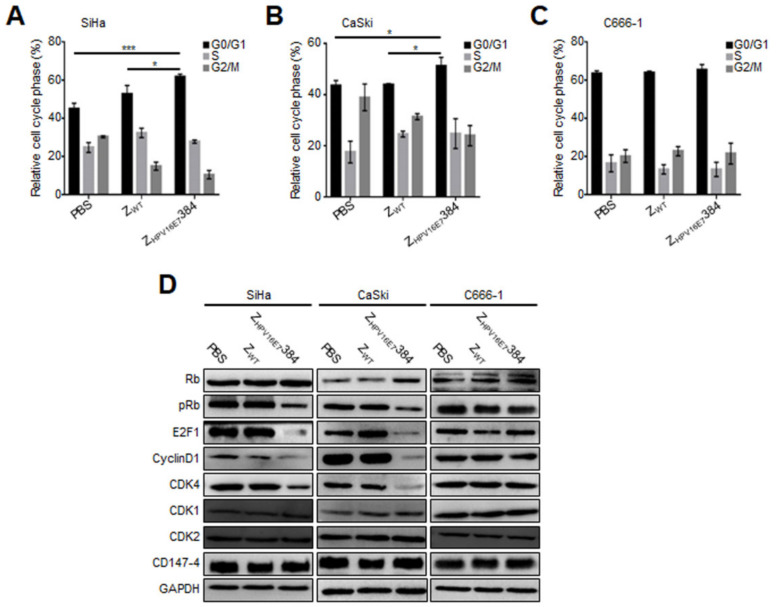
Z_HPV16E7_384 induced G1/S cell cycle arrest by interfering with the E7/Rb/E2F1/cyclin D1/CDK4 pathway. (**A**–**C**) A cell cycle analysis was performed to evaluate the effect of Z_HPV16E7_384 on the cell cycle. Cells treated with Z_WT_ or PBS were used as the negative controls. (**D**) Cell cycle related proteins in cells with the same treatment as in (**A**–**C**) were analyzed by Western blot. The data shown are representative of three independent experiments. All experiments were performed in triplicate and data are expressed as the means ± SD (*n* = 3). * *p* < 0.05, *** *p* < 0.001.

To explore the mechanism of Z_HPV16E7_384 inhibiting the target cell proliferation, we analyzed the effect of Z_HPV16E7_384 on the cell cycle by flow cytometry analysis. As shown in Figure 3A–C, compared to PBS and Z_WT_, Z_HPV16E7_384 treatment resulted in a significant G1/S cell cycle arrest in SiHa and CaSki cells, but not in the C666-1 cells.

Western blot was then performed to determine the expression of the cellular proteins that were involved in Z_HPV16E7_384-induced G1/S cell cycle arrest. As shown in Figure 3D, compared with the control cells, the cell cycle related proteins including phosphorylated retinoblastoma protein (pRb), E2F1, cyclin D1, and CDK4 in the Z_HPV16E7_384-treated SiHa and CaSki cells were significantly downregulated whereas Rb was upregulated. However, other cell cycle related proteins including CDK1, CDK2, and CD147-4 were not affected by Z_HPV16E7_384 treatment. Taken together, these results indicate that Z_HPV16E7_384 was internalized specifically into the target cells, leading to G1/S cell cycle arrest.

### 3.5. The HPV16E7 Protein Played an Important Role during the Z_HPV16E7_384 Internalization

**Figure 4:**
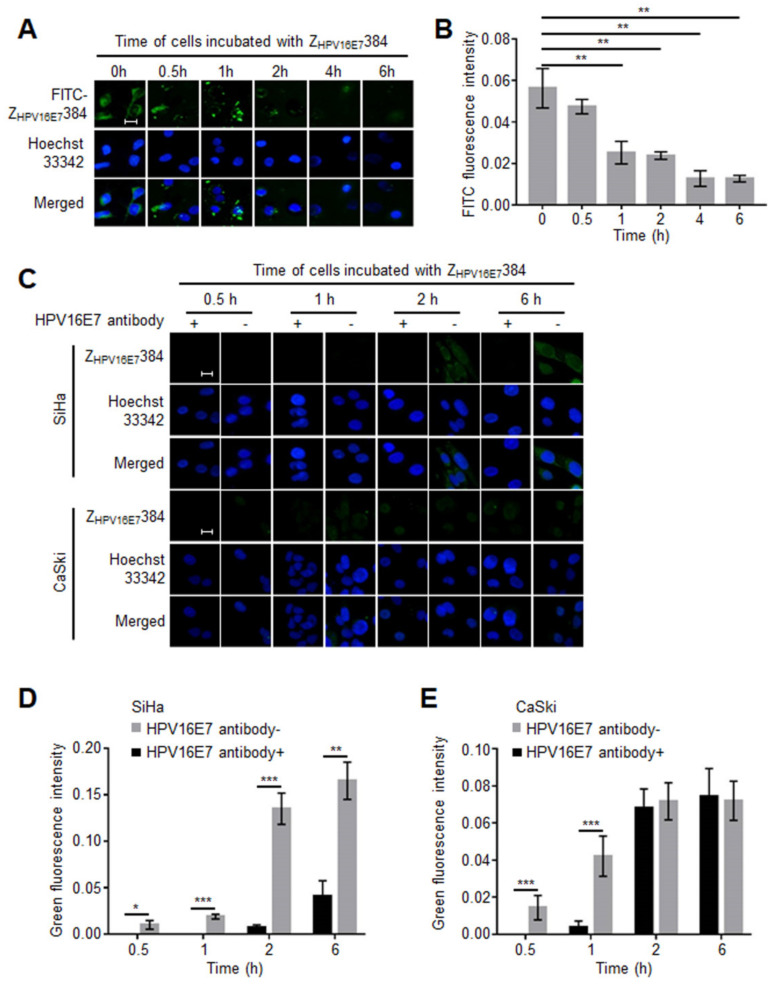
HPV16 E7 was involved in Z_HPV16E7_384 internalization. (**A**) A free affibody blocking assay was performed to evaluate whether the Z_HPV16E7_384 internalization depended on some cellular adapter proteins. The SiHa cells were pre-incubated with free Z_HPV16E7_384 for different time periods and then incubated with FITC-Z_HPV16E7_384 (Green). Cell nuclei were stained with Hoechst33342 (blue). Scale bar = 20 μm. (**B**) The intensity of green fluorescence in (**A**) was analyzed. (**C**) A HPV16E7 antibody blocking assay was performed to evaluate whether the HPV16E7 protein played a role during Z_HPV16E7_384 internalization. The SiHa and CaSki cells were incubated with the rabbit anti-HPV16E7 polyclonal antibody and then with Z_HPV16E7_384 for different time periods. Z_HPV16E7_384 was analyzed by IFA (green). Cell nuclei were counterstained with Hoechst33342 (blue). Cells incubated only with Z_HPV16E7_384 were used as the controls. Scale bar = 20 μm. (**D**,**E**) The intensity of green fluorescence in (**C**) was analyzed. The data shown are representative of three independent experiments. All experiments were performed in triplicate and data are expressed as the means ± SD (*n* = 3). * *p* < 0.05, ** *p* < 0.01, *** *p* < 0.001.

To explore the mechanism of the Z_HPV16E7_384 internalization, a free Z_HPV16E7_384 blocking assay was performed. Both Z_HPV16E7_384 and Z_WT_ were labeled with FITC (FITC-Z_HPV16E7_384 and FITC-Z_WT_), respectively. As shown in Figure S2, FITC-Z_HPV16E7_384, not FITC-Z_WT_, specifically bound to the SiHa cells while it did not bind to the C666-1 cells, indicating that FITC-labelling did not affect the targeting specificity of Z_HPV16E7_384. Then, the SiHa cells pre-incubated with unlabeled Z_HPV16E7_384 (free Z_HPV16E7_384) for different time periods were incubated with FITC-Z_HPV16E7_384 for 6 h. As shown in Figure 4A,B, the amount of FITC-Z_HPV16E7_384 binding to the target cells gradually decreased as the time increased for incubation with free Z_HPV16E7_384. From 1 h, a significantly different fluorescence intensity was observed between cells blocked by free Z_HPV16E7_384 and the untreated cells.

According to the results of the free affibody blocking assay, we speculated that there were some adapter proteins involved in the Z_HPV16E7_384 internalization. Two experiments were then carried out to figure out what kind of adapter proteins might be involved. As reported previously, the affibody was derived from SPA [22], which is known to bind to the Fc region of IgG. Our sequence analysis results showed that the amino acid sequence of Z_HPV16E7_384 was highly similar to the B domain in the IgG binding region of SPA (Figure S3A). Thus, it is possible that Z_HPV16E7_384 could bind to IgG, which may be involved in the Z_HPV16E7_384 internalization because a large number of IgGs are present in the FBS-supplemented cell culture medium. To verify this speculation, the SiHa cells were respectively cultured in the medium with or without FBS supplement. Then, the cells grown in the two media were all incubated with Z_HPV16E7_384 for 6 h, followed by IFA analysis. However, the results showed no difference in Z_HPV16E7_384 internalization between the two groups (Figure S3B,C). In a previous study, HPV16E7 was reported to distribute on the cell membrane [21]. We repeated the study, confirming that HPV16E7 could localize at the cell membrane of HPV16 positive cells (Figure S4). We also proved that Z_HPV16E7_384 could specifically bind to HPV16E7 both in vivo and in vitro [6]. Thus, we reasonably speculated that HPV16E7 proteins at the cell membrane might recruit Z_HPV16E7_384 to assist in its internalization. To verify this speculation, the SiHa and CaSki cells were incubated with a rabbit anti-HPV16E7 polyclonal antibody and then with Z_HPV16E7_384 for 0.5, 1, 2, and 6 h. All of the cells were analyzed by IFA. Cells incubated only with Z_HPV16E7_384 were used as controls. As shown in Figure 4C–E, the amount of Z_HPV16E7_384 binding to the target cells was significantly lower in the SiHa cells incubated with anti-HPV16E7 polyclonal antibody than that in the control cells. Similar results were also observed in the CaSki cells incubated with Z_HPV16E7_384 for 0.5 and 1 h. These results suggest that Z_HPV16E7_384 could be internalized specifically into target cells by binding partially to the HPV16E7 on the target cell surface.

### 3.6. Dynamin and Caveolin-1 Were Indispensable for the Z_HPV16E7_384 Internalization

**Figure 5:**
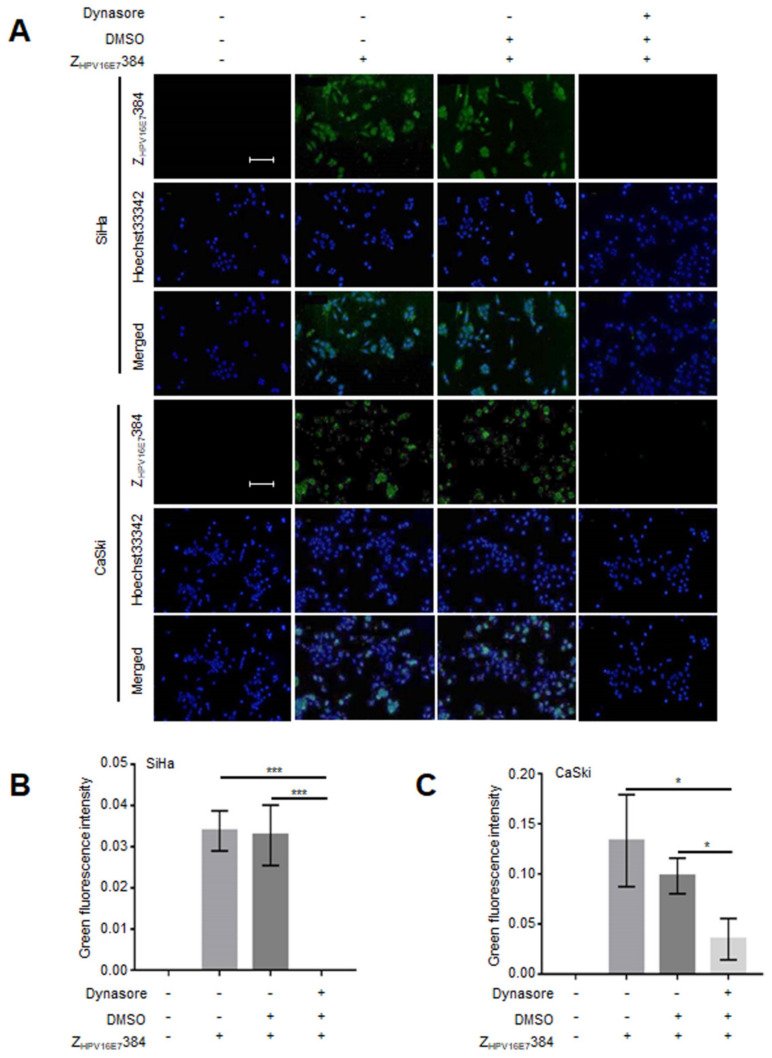
The Z_HPV16E7_384 internalization was dependent on Dynamin. (**A**) The SiHa and CaSki cells that were first treated with the dynamin inhibitor Dynasore in DMSO and then incubated with Z_HPV16E7_384 were analyzed by IFA (green). Cell nuclei were counterstained with Hoechst33342 (blue). Scale bar = 100 μm. (**B**,**C**) The intensity of the green fluorescence in (**A**) was analyzed. The data shown are representative of three independent experiments. All experiments were performed in triplicate and data are expressed as the means ± SD (*n* = 3). * *p* < 0.05, *** *p* < 0.001.

**Figure 6:**
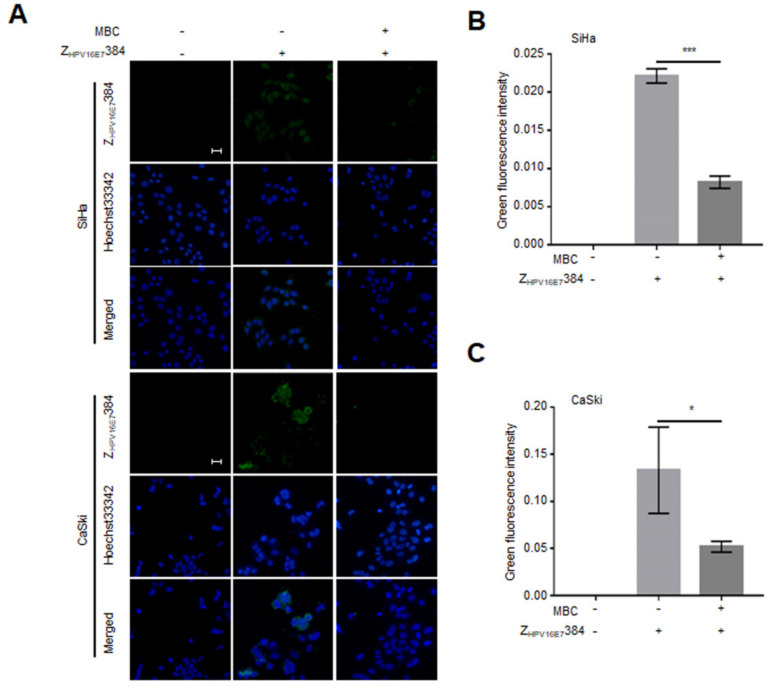
The Z_HPV16E7_384 internalization was dependent on caveolin-1. (**A**) The SiHa and CaSki cells that were first treated with the caveolin-1 mediated endocytosis inhibitor MBC and then incubated with Z_HPV16E7_384 were analyzed by IFA (green). Cell nuclei were stained with Hoechst33342 (blue). Scale bar = 20 μm. (**B**,**C**) The intensity of the green fluorescence in (**A**) was analyzed. The data shown are representative of three independent experiments. All experiments were performed in triplicate and data are expressed as means ± SD (*n* = 3). * *p* < 0.05, *** *p* < 0.001.

To explore the possible Z_HPV16E7_384 internalization pathway, the SiHa and CaSki cells were first treated with the CDE inhibitor CPZ, or the DDE inhibitor Dynasore, or the micropinocytosis inhibitor Wortmannin, or the CaDE inhibitor MBC, and then incubated with Z_HPV16E7_384. As shown in the IFA results, Dynasore or MBC treatment significantly inhibited the Z_HPV16E7_384 internalization (Figure 5 and Figure 6) whereas CPZ and Wortmannin had no effect on the Z_HPV16E7_384 internalization (Figures S5 and S6).

**Figure 7:**
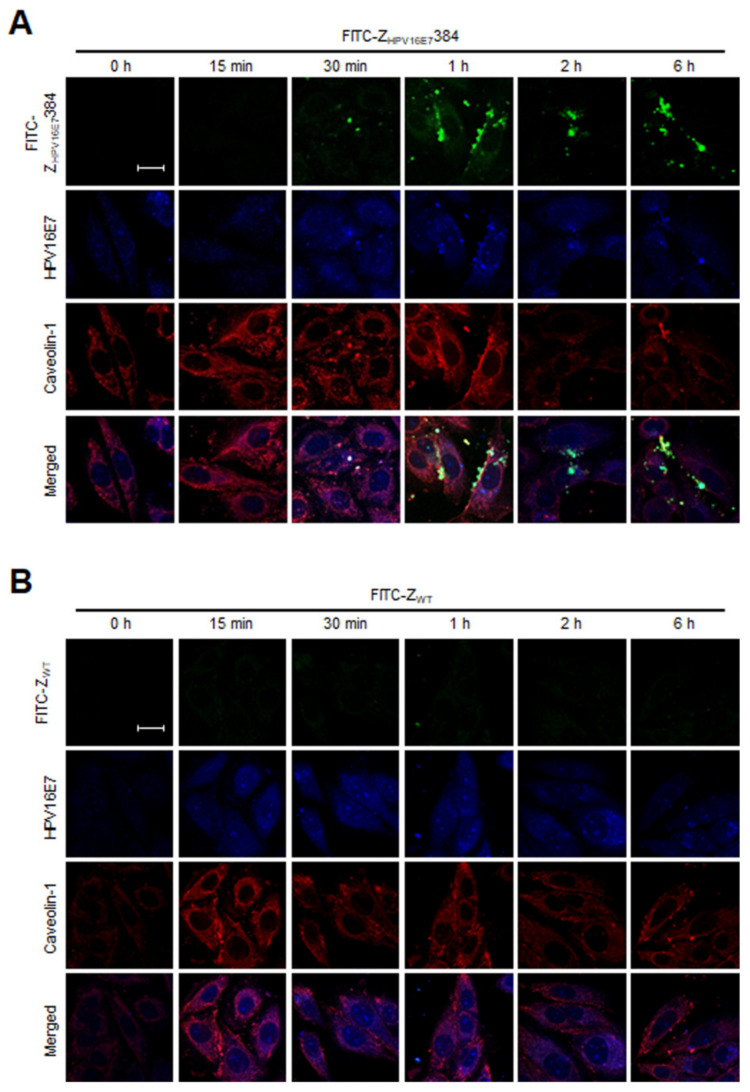
Z_HPV16E7_384, HPV16E7, and caveolin-1 were co-localized during the process of Z_HPV16E7_384 internalization. (**A**) The SiHa cells were incubated with FITC-conjugated Z_HPV16E7_384 (green) for different time periods. Then, all cells were analyzed by confocal fluorescence microscopy using goat anti-caveolin-1 polyclonal antibody and rabbit anti-HPV16E7 polyclonal antibody as the primary antibodies and using the CY3-conjugated donkey anti-goat IgG polyclonal antibody (red) and the ALexar fluor 350-conjugated goat anti-rabbit IgG polyclonal antibody (blue) as the secondary antibodies, respectively. The merged images showed the co-localization of Z_HPV16E7_384, HPV16E7, and caveolin-1. Scale bar = 20 μm. (**B**) The SiHa cells incubated with FITC-conjugated Z_WT_ for different time periods were used as the negative controls. The data shown are representative of three independent experiments.

To further confirm the dependence of caveolin-1 during Z_HPV16E7_384 internalization, the co-localization of Z_HPV16E7_384, HPV16E7, and caveolin-1 was analyzed by confocal fluorescence microscopy. As shown in Figure 7, after the SiHa cells were incubated with FITC-Z_HPV16E7_384 for 30 min, the co-localization of Z_HPV16E7_384, HPV16E7, and caveolin-1 was observed. With the increase in the time that the cells were incubated with FITC-Z_HPV16E7_384, the co-localization became more and more obvious. However, the co-localization was not observed in the SiHa cells incubated with FITC-Z_WT_.

**Figure 8:**
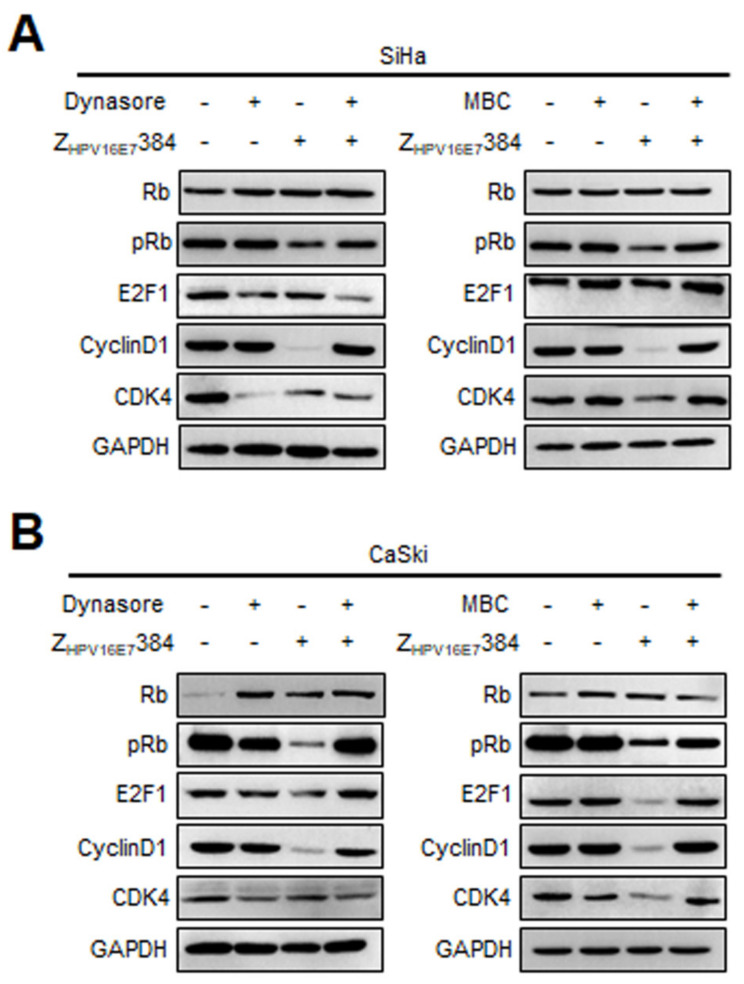
Dynasore and MBC reversed the effect of Z_HPV16E7_384 on cell cycle-related proteins. (**A**) The SiHa and (**B**) CaSki cells were respectively pre-treated with Dynasore and MBC and then incubated with Z_HPV16E7_384. The cell cycle-related proteins were then analyzed by Western blot. The SiHa and CaSki cells only treated with PBS, the inhibitors, or Z_HPV16E7_384 were used as the controls.

To further verify the endocytic pathway of Z_HPV16E7_384, the SiHa and CaSki cells were respectively pre-treated with Dynasore or MBC and then incubated with Z_HPV16E7_384, followed by Western blot analysis. The SiHa and CaSki cells only treated with PBS, the inhibitors, or Z_HPV16E7_384 were used as the controls. As shown in Figure 8A, in the SiHa cells, compared with only the Z_HPV16E7_384 treatment, pre-treatment with Dynasore rescued the downregulation of pRb and cyclin D1 induced by Z_HPV16E7_384 treatment. Similarly, compared with only the Z_HPV16E7_384 treatment, pre-treatment with MBC rescued the downregulation of pRb, E2F1, cyclin D1, and CDK4 induced by Z_HPV16E7_384 treatment. Similar results were observed in the CaSki cells (Figure 8B). Taken together, these results indicated that Z_HPV16E7_384 internalization at least partially depended on dynamin- and caveolin-1 mediated endocytosis. 

## 4. Discussion

Affibodies have been developed as an alternative to antibodies for biotechnological applications over the last two decades [1,23]. Some therapeutic affibodies have now entered preclinical and clinical trials for cancer treatment [5]. Recently, several affibodies using intracellular proteins as targets show great potential in cancer and other disease treatment [6,7,8,9,10,11]. We have previously developed the affibody Z_HPV16E7_384 as a cervical cancer therapeutic agent [6,7]. In this study, we investigated the internalization specificity of Z_HPV16E7_384 and its underlying mechanisms entering two target cervical cancer cells: SiHa and CaSki cells. The two target cells were HPV16 positive [24]. As a result, we confirmed that Z_HPV16E7_384 internalized specifically into the target cells, consistent with our previously published results [6,7] and a recent study that the Z_HPV16E7_384 affibody had intense and specific staining for the HPV16 E7 oncoprotein in the cervical cancer biopsies [25]. 

Furthermore, we found that Z_HPV16E7_384 significantly inhibited the proliferation of the target cancer cells, which explains to some extent the anti-cervical cancer efficacy of Z_HPV16E7_384 in tumor-bearing nude mice reported in our previous study [7]. The Z_HPV16E7_384 inhibition in target cell proliferation could be partially attributed to G1/S cell cycle arrest. It is well-established that the transition of different phases during the cell cycle is regulated by specific cyclin–CDK complexes [26]. Rb downregulates the expression of cyclins and CDKs by binding with transcription factor E2Fs, subsequently suppressing the entry of cells into the S phase [27,28,29]. However, Rb can be phosphorylated by cyclin–CDK complexes, leading to the partial release of Rb from the E2Fs to promote cell cycle progression from G1 to the S phase [30,31,32,33,34,35]. In the HPV16 positive cervical cancer cells, HPV16 E7 binds to and degrades Rb [36], while Z_HPV16E7_384 binds specifically with HPV16 E7 [6], which might inhibit the degradation of Rb. As a result, we observed here that the level of Rb was significantly higher in the target cells treated with Z_HPV16E7_384 than that in the control cells. On the other hand, the levels of pRb, E2F1, cyclin D1, and CDK4 were significantly reduced in the Z_HPV16E7_384-treated target cells. In addition, our Western blotting analysis showed that Z_HPV16E7_384 treatment did not inhibit the expression of three other cell cycle related proteins: CD147-4, CDK1, and CDK2 [37,38], implying that Z_HPV16E7_384 specifically inhibited the expression of the cell cycle proteins cyclin D1 and CDK4 in the target cancer cells. Cyclin D1 and CDK4 are the key cell cycle regulators, which regulate the timing of the events in the cell cycle [39,40]. Z_HPV16E7_384 treatment inhibited the expression of cyclin D1 and CDK4 to disrupt their normal cell cycle regulatory functions, leading to the cell cycle arrest at the G1/S phase in target cancer cells, which is more likely through the coordinated interactions between the cyclin D1/CDK4 and Rb/E2F1 complexes. Thus, the results suggest that (1) Z_HPV16E7_384 bound to HPV16 E7 to prevent the release of Rb from E2Fs and its degradation after specifically being internalized into the target cells and (2) Rb bound to E2F1 to form a Rb/E2F1 complex, which interacted with the cyclin D1/CDK4 complex to inactivate the E2F1 transcription factor, leading to cell proliferation inhibition through the G1/S cell cycle arrest. The interactions of Rb/E2F1 with cyclin D1/CDK4 also inhibited the phosphorylation of Rb to stabilize the Rb/E2F1 complex, promoting the G1/S cell cycle arrest. Consequently, Z_HPV16E7_384 affibody treatment inhibited the proliferation of the target cancer cells.

We have previously demonstrated that Z_HPV16E7_384 binds specifically to the HPV16 E7 oncoprotein in HPV 16 positive cervical cancer cells [6,7]. However, HPV16 E7 is generally localized in the nucleus and cytoplasm [41,42]. A question that has been raised is how Z_HPV16E7_384 enters the target cells to interact with the E7 protein. It is well-known that endocytosis plays crucial roles in the uptake of extracellular nutrients, hormones, and cargoes [43]. Thus, Z_HPV16E7_384 might enter the target cells through a crucial endocytosis pathway. Clathrin and dynamin are the two key proteins that play the most important roles in ensuring the successful completion of endocytosis. Based on clathrin, endocytosis has two pathways: clathrin-dependent endocytosis (CDE) and clathrin-independent endocytosis (CIE) [43]. Furthermore, CIE can be further divided into caveolin-dependent endocytosis (CaDE) and caveolin-independent endocytosis (CaIE) [43]. Based on dynamin, endocytosis also has two pathways: dynamin dependent endocytosis (DDE) and dynamin independent endocytosis (DIE) [44]. DDE includes CDE, CaDE, and micropinocytosis [44], while DIE only includes CaIE [45]. In this study, we used four endocytosis-related protein inhibitors: Dynasore, MBC, CPZ, and Wortmannin to treat the target cells, respectively, to investigate whether Z_HPV16E7_384 internalized into the target cells via endocytosis, or just attached on the cell surface. Our results showed that two inhibitors (Dynasore and MBC), not the other two inhibitors (CPZ and Wortmannin), significantly constrained Z_HPV16E7_384 internalization. We also observed that Z_HPV16E7_384 co-localized with HPV16 E7 and caveolin-1 in the target cells. Thus, the results excluded the possibility of Z_HPV16E7_384 attachment only on the surface of the target cell, revealing that Z_HPV16E7_384 might enter the target cell through the cellular endocytosis pathway. Because Dynasore is an inhibitor of dynamin [46] and MBC is an inhibitor of caveolin-1 [47,48], the results thus suggest that dynamin and caveolin-1 played important roles in assisting Z_HPV16E7_384 endocytosis, which was further confirmed by the results that Dynasore or MBC treatment reversed the effect of Z_HPV16E7_384 on the cell cycle-related proteins. Despite the inherent lack of specificity of these two inhibitors [49,50,51], both affected the internalization of Z_HPV16E7_384 similarly, so it remains likely that dynamin and caveolin-1 dependent endocytosis is important for the function of Z_HPV16E7_384. To provide more evidence, the knockdown of dynamin or caveolin-1, respectively, was also attempted but resulted in substantial cell death (data not shown). In addition, HPV16 E7 could also localize at the cell membrane [21], which was confirmed by our results. In this regard, HPV16E7 on the cell surface might serve as a receptor for the endocytosis of Z_HPV16E7_384, which was then verified by the HPV16 E7 antibody blocking assay.

## 5. Conclusions

**Figure 9:**
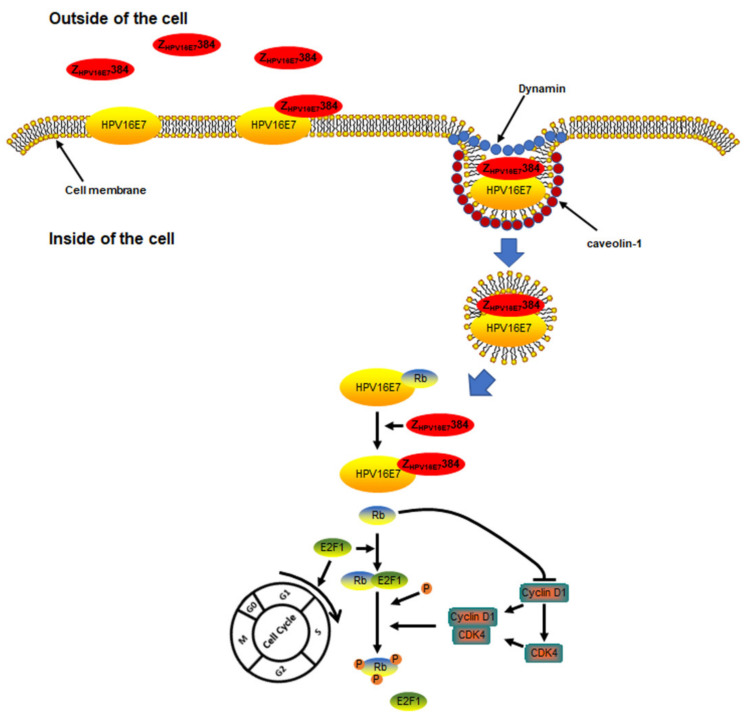
A schematic model to summarize the internalization pathway of Z_HPV16E7_384 and its effect on the cell proliferation related pathway.

In conclusion, our results appear to show that Z_HPV16E7_384 specifically internalized into the target cells depending on the endocytosis, with two proteins (dynamin and caveolin-1) that mediated the endocytosis processes and that Z_HPV16E7_384 inhibited target cell proliferation by at least partially interrupting the E7/Rb/E2F1/cyclin D1/CDK4 pathway (Figure 9). Further studies need to be performed to confirm the conclusion. Possibly, the findings in this study provide a rationale of using Z_HPV16E7_384 to conduct a clinical trial for target therapy in cervical cancer.

## Data Availability

Not applicable.

## Supplementary Materials

The following supporting information can be downloaded at: https://www.mdpi.com/article/10.3390/biom12081114/s1, Figure S1: Preparation of Z_HPV16E7_384; Figure S2: FITC-labelled Z_HPV16E7_384 could bind specifically to the HPV16 positive cells; Figure S3: The internalization of Z_HPV16E7_384 was independent of IgG; Figure S4: HPV16E7 localized on the cell membrane of the SiHa cells; Figure S5: The CDE inhibitor CPZ had no effect on the internalization of Z_HPV16E7_384; Figure S6: The micropinocytosis inhibitor Wortmannin had no effect on the internalization of Z_HPV16E7_384.
