# Supplementary material for: The HPV16E7 Affibody as a Novel Potential Therapeutic Agent for Treating Cervical Cancer Is Likely Internalized through Dynamin and Caveolin-1 Dependent Endocytosis
Source: Biomolecules. 2022 Aug 12;12(8):1114. doi: 10.3390/biom12081114 (PMC9405713; doi:10.3390/biom12081114)
Supplement: Supplementary file 1 [file biomolecules-12-01114-s001.zip › biomolecules-1796947-supplementary.pdf]

The HPV16E7 Affibody as a Novel Potential Therapeutic Agent for Treating Cervical Cancer Is Likely Internalized through Dynamin and Caveolin-1 Dependent Endocytosis

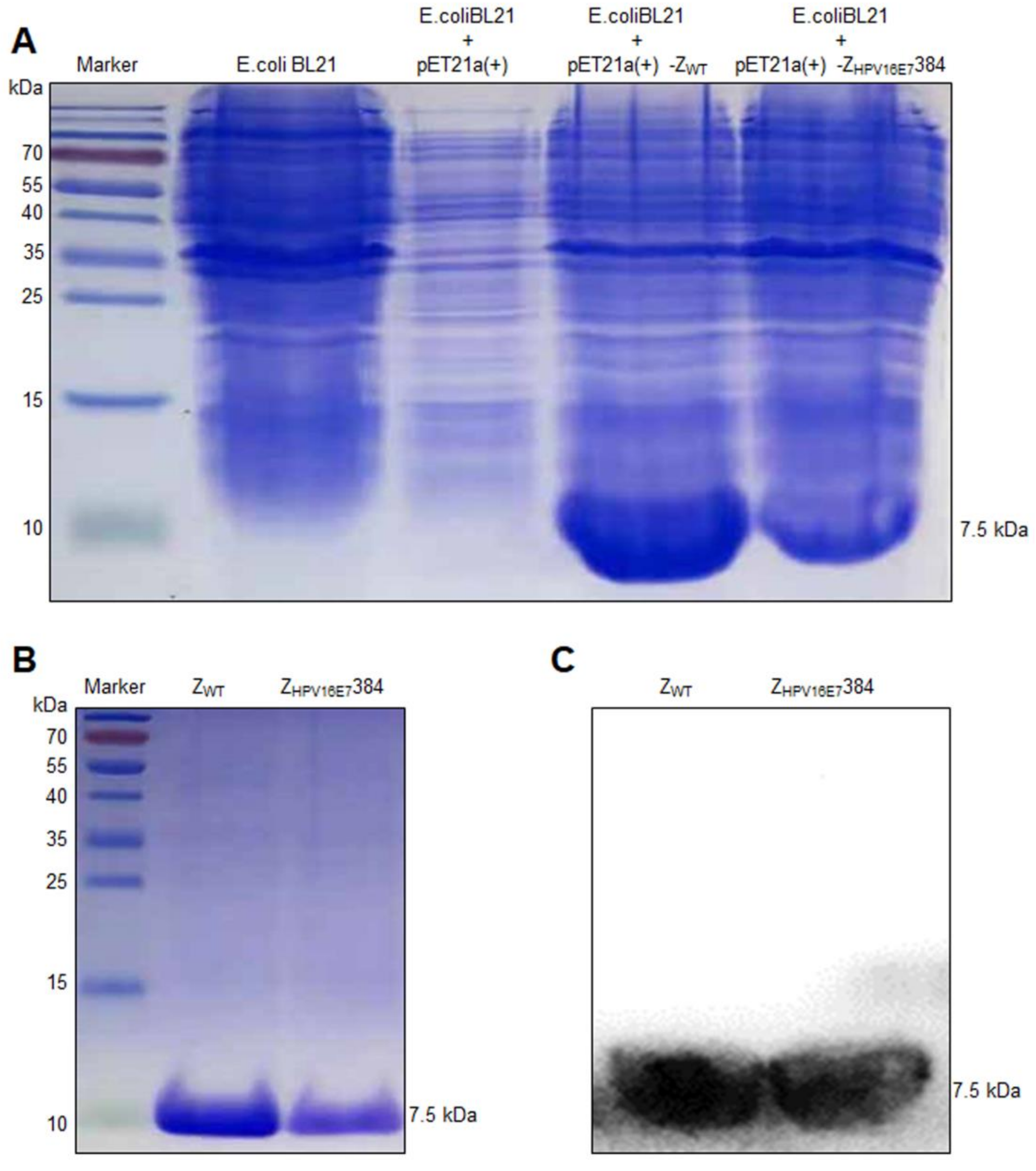

Figure S1. Preparation of $Z_{HPV16E7}$384. A, The E.coli BL21 (DE3) transformed with plasmid pET21a(+)-$Z_{HPV16\ E7}$384 was used to express and purify $Z_{HPV16E7}$384 protein. B, The purified $Z_{HPV16E7}$384 was analyzed by SDS-PAGE. C, The purified $Z_{HPV16E7}$384 was analyzed by western blot.

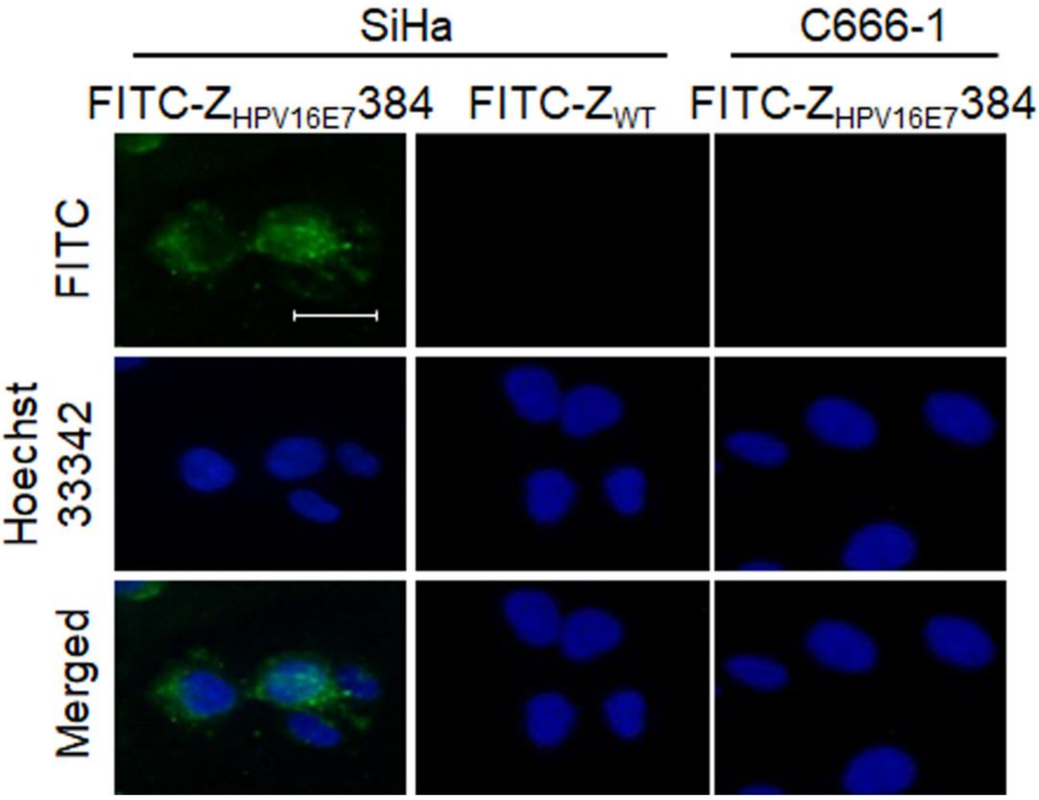

Figure S2. FITC-labelled $Z_{HPV16E7}$384 could specifically bind to HPV16 positive cells. SiHa cells were firstly incubated with FITC-$Z_{HPV16E7}$384 (Green) and then were stained with Hoechst33342 (Blue). SiHa cells incubated with FITC-Z_{WT} and C666-1 cells incubated with FITC-$Z_{HPV16E7}$384 were used as negative controls. All cells were analyzed by a confocal fluorescence microscope. Scale bar = 20 μm.

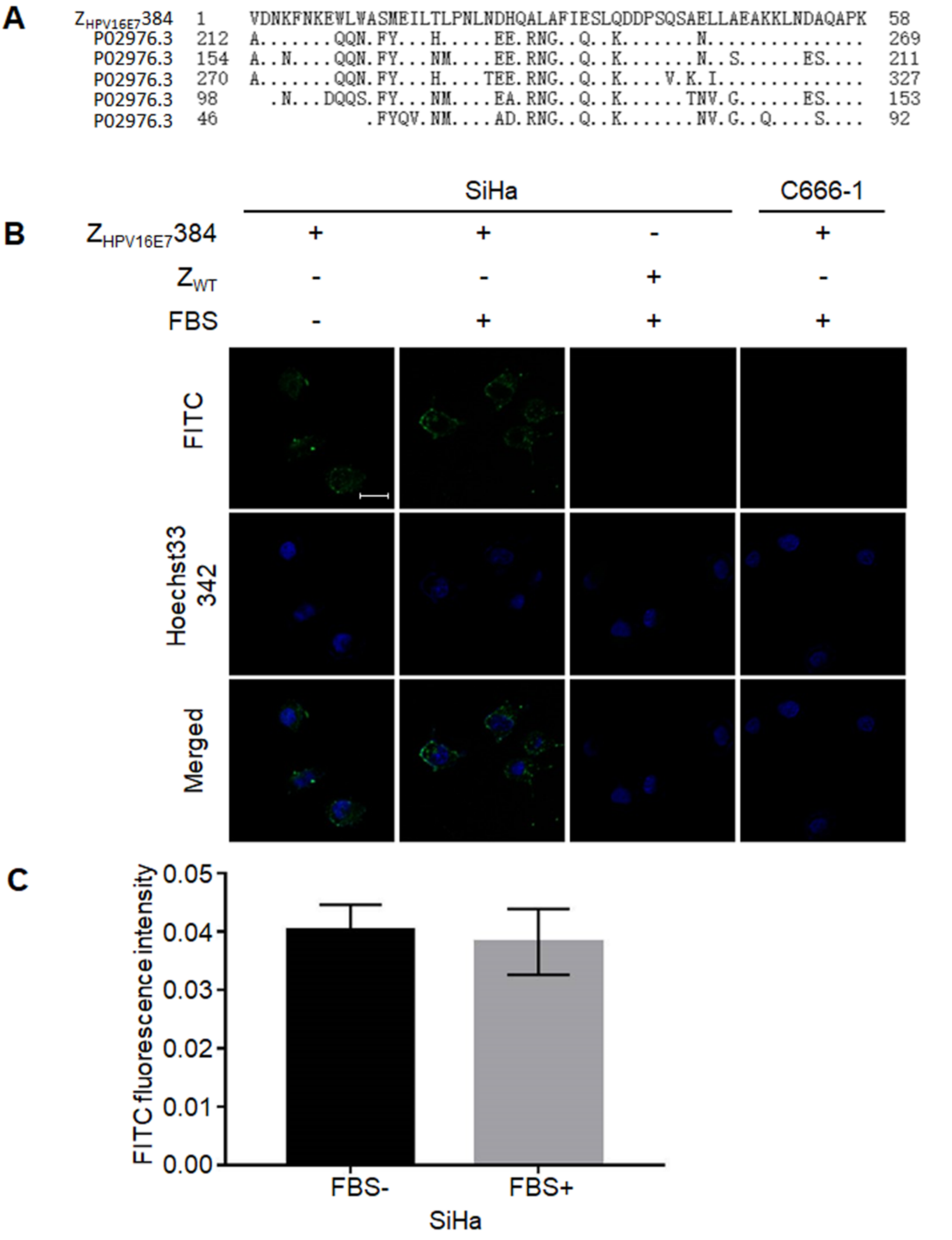

Figure S3. The internalization of $Z_{HPV16E7}384$ was independent of IgG. A, The amino acid sequence of $Z_{HPV16E7}384$ was compared with that of the B domain in the IgG binding region of SPA. The same amino acids between $Z_{HPV16E7}384$ and SPA were shown as dots. B, SiHa cells incubated with $Z_{HPV16E7}384$ diluted in medium supplemented with or without 10% fetal bovine serum for 6 h were

used as tested cells. SiHa cells incubated with Z_{WT} and C666-1 cells incubated with $Z_{HPV16E7}384$ were used as negative controls. All cells were analyzed by the indirect immunofluorescence assay using mouse anti-His tag monoclonal antibody as the primary antibody and FITC-conjugated goat anti-mouse IgG polyclonal antibody as the secondary antibody (Green). Cell nuclei were stained with Hoechst33342 (Blue). Scale bar = 20 μm. C, The intensity of green fluorescence in Fig. S3B was analyzed. The data shown are representative of three independent experiments.

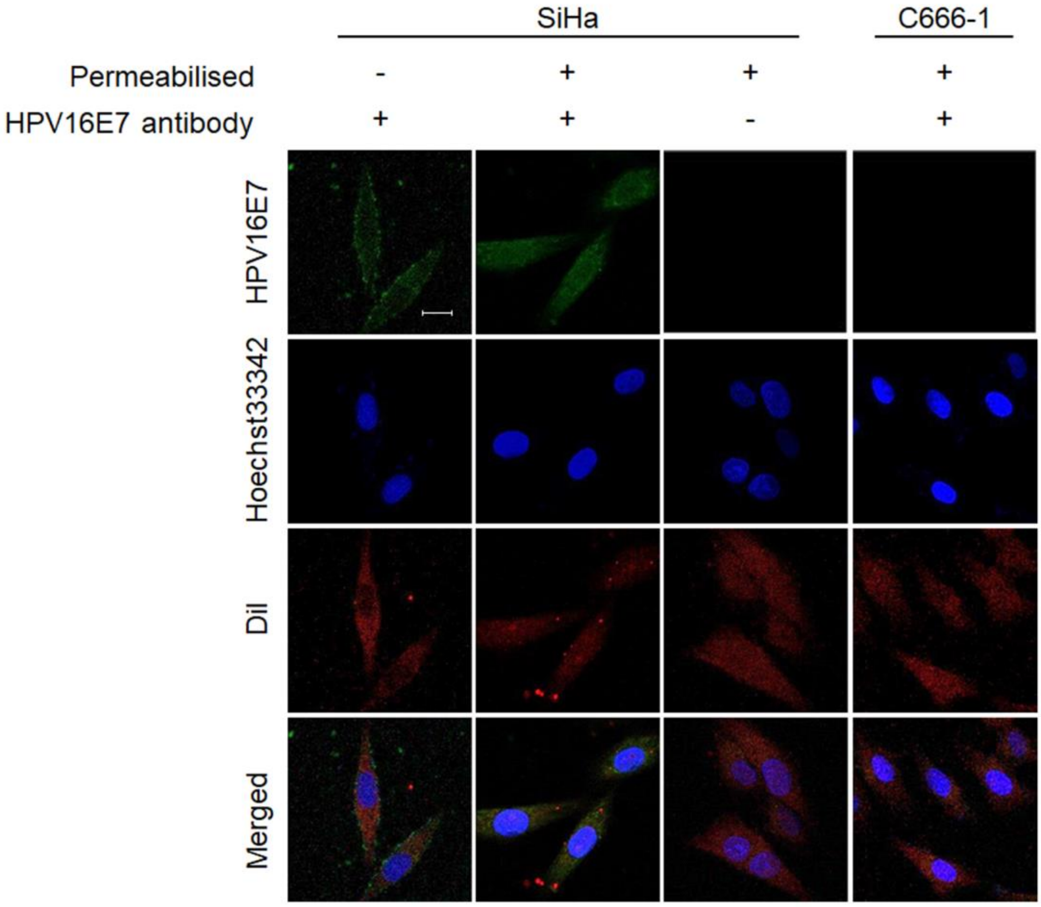

Figure S4. The distribution of HPV16E7 on the cell membrane of SiHa cells was determined by an indirect immunofluorescence assay. Live SiHa cells were firstly incubated with rabbit anti-HPV16E7 polyclonal antibody for 1.5 h and then were fixed and incubated with FITC-conjugated goat anti-rabbit IgG polyclonal antibody as the secondary antibody (Green). Cell nuclei were stained with Hoechst33342 (Blue). Cell membranes were stained with $DiIC_{18}(3)$ (Red). Scale bar = 20 μm. The

fixed and permeabilized SiHa cells incubated with primary and secondary antibodies were used as a positive control. The fixed and permeabilized SiHa cells incubated with only secondary antibodies and the fixed and permeabilized C666-1 cells incubated with primary and secondary antibodies were used as negative controls. The data shown are representative of three independent experiments.

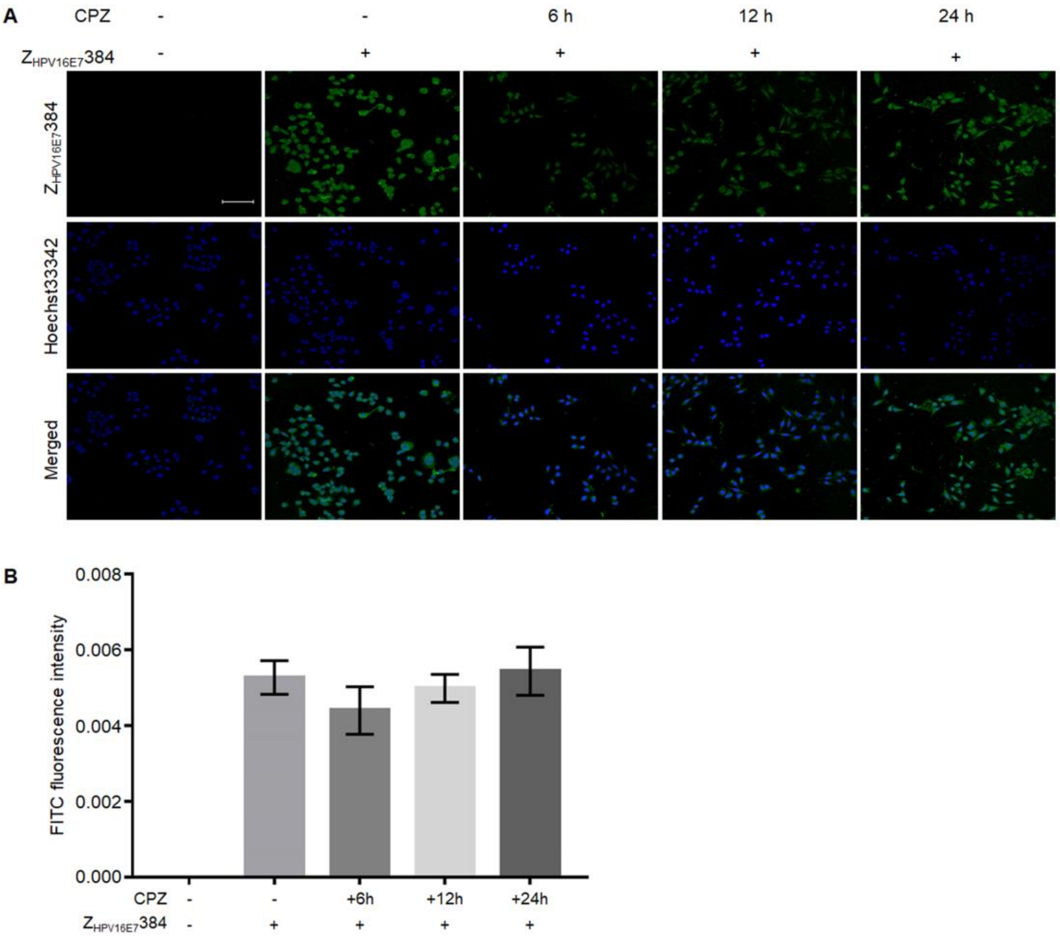

Figure S5. The CME inhibitor CPZ had no effect on the internalization of $Z_{HPV16E7}$384. A, SiHa cells were firstly treated with 30 μM of CPZ for different time periods and then were incubated with 50 μM of $Z_{HPV16E7}$384 for 6 h. The internalized $Z_{HPV16E7}$384 were detected by an indirect immunofluorescence assay (Green). Cell nuclei were stained with Hoechst33342 (Blue). SiHa cells without any treatment were used as a negative control while SiHa cells only incubated with 50 μM of $Z_{HPV16E7}$384 were used as a positive control. B, The intensity of green fluorescence in Fig. S5A was analyzed. The data shown are representative of three independent experiments.

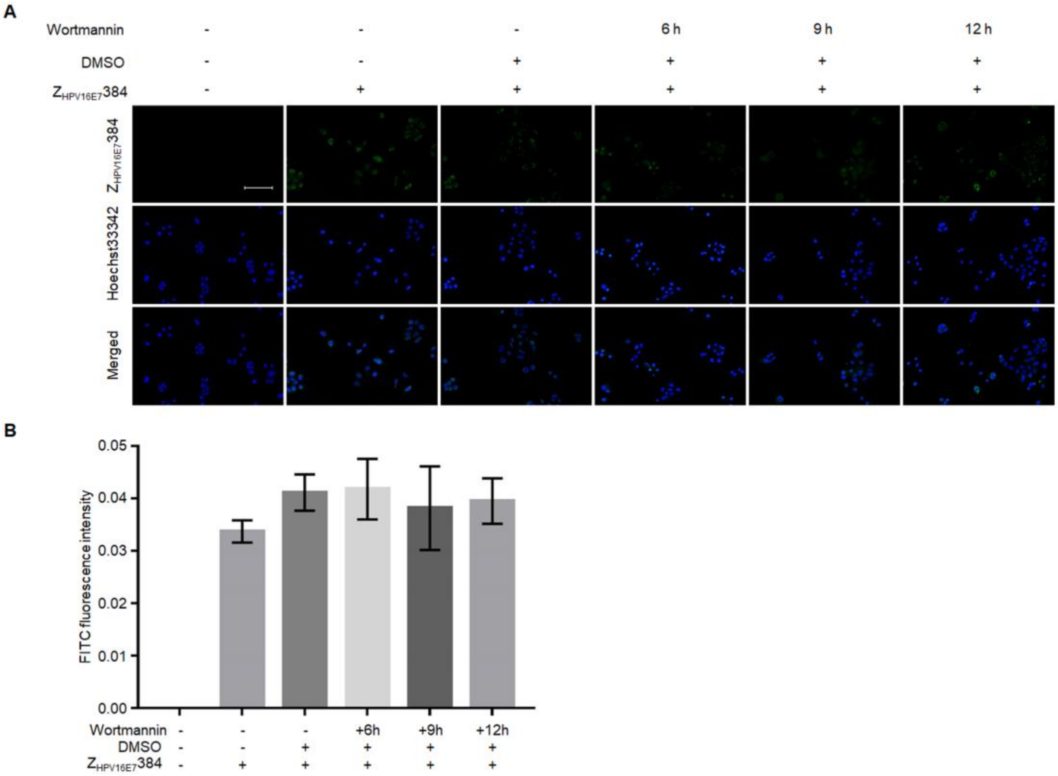

Figure S6. The micropinocytosis inhibitor Wortmannin had no effect on the internalization of $Z_{HPV16E7}$384. A, SiHa cells were firstly treated with 30 μM of Wortmannin in DMSO for different time periods and then were incubated with 50 μM of $Z_{HPV16E7}$384 for 6 h. The internalized $Z_{HPV16E7}$384 were detected by an indirect immunofluorescence assay (Green). Cell nuclei were stained with Hoechst33342 (Blue). SiHa cells only incubated with 50 μM of $Z_{HPV16E7}$384 were used as a positive control while SiHa cells without any treatment were used as a negative control. SiHa cells treated with DMSO were used as a mock control. B, The intensity of green fluorescence in Fig. S6A was analyzed. The data shown are representative of three independent experiments.
